# Epidemiological characteristics of myopia among school-age children before, during, and after the COVID-19 pandemic: a cohort study in Shenzhen, China

**DOI:** 10.3389/fmed.2024.1368219

**Published:** 2024-08-30

**Authors:** Jingfeng Mu, Haoxi Zhong, Mingjie Jiang, Weihua Yang

**Affiliations:** Shenzhen Eye Hospital, Jinan University, Shenzhen Eye Institute, Shenzhen, China

**Keywords:** myopia, epidemiology, school-aged children, COVID-19, cohort study

## Abstract

**Objectives:**

To evaluate the epidemiological characteristics of myopia among school-aged children before, during, and after the coronavirus disease (COVID-19) pandemic.

**Methods:**

A total of 848,697 students aged 6–15 years from 786 primary and secondary schools in Shenzhen, China, were randomly selected as research subjects. We conducted annual myopia screenings from 2019 to 2022. 2019 was considered before the COVID-19 pandemic, 2020 as during the pandemic, and 2021 and 2022 as after the pandemic. Demographic characteristics, visual acuity, and spherical equivalent refraction (SE) were collected.

**Results:**

During the 4-year follow-up period, the uncorrected visual acuity (UCVA) of the study subjects progressed following a trend of −0.18 ± 0.30D (−0.17 ± 0.29D for boys, −0.21 ± 0.32Dfor girls) (*p* < 0.001). Those students who were in grade 4 aged 9–10 years at the baseline examination showed the greatest decline in visual acuity (0.23). The SE of the study subjects progressed following a trend of −1.00 ± 1.27D (−0.96 ± 1.25D for boys, −1.05 ± 1.31D for girls) (*p* < 0.001). The students who were in grade 5 aged 10–11 years at the baseline examination showed the greatest decline in SE (1.15D ± 1.22, *p* < 0.001). The prevalence of myopia (UCVA<5.0 and SE < –0.50D of any eye) increased by 28.2% (27.0% for boys and 29.8% for girls). Those students who were in grade 2 aged 7–8 years at the baseline examination showed the greatest increase in myopia prevalence (37.6%, *p* < 0.001). During the COVID-19 pandemic, the subjects’ visual acuity and SE measurements decreased by −0.05 ± 0.19 (*p* < 0.001) and − 0.36 ± 0.89D (*p* < 0.001) respectively, and the prevalence of myopia increased by 11.3% (10.6% for boys and 12.2% for girls) (*p* < 0.001). The 3-year cumulative incidence of myopia for non-myopic grade 1 aged 6–7 years students with baseline SE of ≥1.00D, ≥ 0.50D and < 1.00D, ≥0D and < 0.50D, and ≥ −0.50D and < 0D were 6.8, 24.8, 39.0, and 48.1%, respectively.

**Conclusion:**

During the COVID-19 pandemic, the SE of school-aged children showed myopic drift and decreased visual acuity. Myopia progressed faster among girls than among boys in the same grades. The risk of myopia among school-aged children persisted even after the home quarantine of the COVID-19 pandemic was lifted.

## Introduction

Myopia is a global public health issue and represents one of the main causes of vision loss. Studies predict that by 2050, half of the world’s population will suffer from myopia and the prevalence of severe myopia will reach 9.8% (1). Myopia has long been an epidemic in Asian populations (2), particularly in China, where its prevalence remains high and is rapidly increasing (3). Myopia, particularly severe myopia, can increase the risk of eye diseases such as retinal detachment, macular degeneration, and glaucoma (4, 5), as well as irreversible visual impairment and blindness (6). Visual impairment poses a huge economic burden across the globe (7), with an annual economic loss of approximately $244 billion worldwide (8).

Since the outbreak of coronavirus disease-19 (COVID-19) in China at the end of 2019, most countries worldwide have controlled its spread by maintaining social distancing, home quarantines, and through other methods (9). China lifted its at-home quarantine program in May 2020. During the COVID-19 pandemic, approximately 278 million primary and secondary school students in China received online education at home (10). One survey found that home quarantine, particularly online learning, led to a reduction in children’s outdoor activities, increased use of electronic devices, and irregular sleep and dietary habits (11, 12). During the COVID-19 pandemic, children spent more time watching electronic screens and less time outdoors (13). Reduced outdoor activity, increased use of electronic devices, and insufficient sleep are known risk factors for the occurrence and progression of myopia (14, 15). Several studies have assessed the development of myopia in children and adolescents before and after the COVID-19 pandemic. The prevalence of myopia among children and adolescents in China’s Chongqing province, for example, increased from 45.3 to 55.4% (16). Similarly, the spherical equivalent refractions (SE) of primary and secondary school students in Wenzhou province progressed from −0.23 D to −0.34 D (17).

Cross-sectional studies have been used to assess the occurrence and development of myopia during the COVID-19 pandemic; however, it has been difficult to assess the impact of the pandemic on myopia. In several cohort studies, the observation time was too short to draw sound conclusions. Cohort studies have important scientific value for revealing the causes of various diseases. When based on large samples and long-term follow-up observations, they can effectively control for various biases, explore the causal relationship between exposure and effects, and provide strong etiological evidence. This study used a large-scale population cohort study to observe a cohort of research subjects for four consecutive years to evaluate the epidemiological characteristics and developmental trends of myopia among school-aged children.

## Methods

### Study design and population

This study selected primary and secondary school students aged 6–15 yeas in Shenzhen to conduct a myopia follow-up cohort study, and longitudinally observed the development trend of myopia in the participants from 2019 to 2022. Using the cluster random sampling method, students from 786 primary and secondary schools in Shenzhen were selected as study participants. Shenzhen has no countryside and is the third most populous city in China. The students recruited for the study were all from urban areas. Students in grades 1–6 aged 6–11 years attending primary schools and students in grades 7–9 aged 12–15 years attending secondary schools were recruited. The included students needed to be able to complete all examinations set for the study, cooperate to complete a 4-year follow-up, and both them and their guardians needed to agree to participate in the study. Students who could not complete the myopia screenings were excluded.

We conducted myopia screenings of the research participants from September to November of every year from 2019–2022. We define the periods as follows: 2019 was considered before the COVID-19 pandemic, 2020 as during the pandemic, and 2021 and 2022 as after the pandemic. A total of 851,891 students participated in myopia screenings for the first time during this study, and 848,697 completed the 4-year follow-up, with a loss-to-follow-up rate of 0.37%. The follow-up results are presented in Table 1. In total, 848,697 primary and secondary school students, including 460,983 boys and 387,714 girls, were enrolled in this study.

**Table 1:** Follow-up of the study participants.

	2019	2022	Rate of loss to follow-up (%)
Grade			
Grade 1	156,024	155,462	0.36
Grade 2	151,172	150,846	0.22
Grade 3	129,281	129,019	0.20
Grade 4	108,089	107,871	0.20
Grade 5	94,930	94,704	0.24
Grade 6	83,947	83,642	0.36
Grade 7	63,300	62,713	0.93
Grade 8	41,638	41,236	0.97
Grade 9	23,510	23,204	1.30
Sex			
Male	462,851	460,983	0.40
Female	389,040	387,714	0.34

This study’s procedure met the requirements of the Declaration of Helsinki and was approved by the Ethics Committee of the Shenzhen Eye Hospital (2023KYYJ047-02). Before participating in the study, the students and their parents/guardians were informed of the research objectives and examination procedures, and informed consent forms were signed by all of the participants’ parents or guardians.

### Data collection

#### Myopia screening

Basic information of participants in the present study including sex, date of birth, school name, and grade were collected. The included research participants were screened for myopia by trained optometrists or ophthalmologists. The screening items included uncorrected visual acuity and refraction tests, and all processes were strictly performed in accordance with the specifications for screening refractive error in primary and secondary school students (WS/T 663–2020). A logarithmic visual acuity chart (Eye Vision 1,603–01) was used to examine uncorrected visual acuity in both eyes. An autorefractor (NIDEK AR-1) was used to check for non-cycloplegic SE. This study relied on the Shenzhen Children and Adolescents Myopia Monitoring Big Data Platform. All screening data was uploaded in real time to the platform for storage and summary.

#### Definition of myopia

Myopia was defined as the uncorrected visual acuity of any eye of the participants <5.0, as well as a non-cycloplegic SE of < −0.50 diopter (D) (18). Myopic students were classified according to the SE values of their right eyes. Mild myopia was defined as −3.00 D ≤ SE < −0.50 D, moderate myopia was defined as −6.00 D ≤ SE < −3.00 D, and severe myopia was defined as SE < −6.00 D.

### Statistical analysis

Statistical analyses were performed using R (version 4.1.0), and the significance level was set at *α* = 0.05. As the binocular visual acuity (*r* = 0.833, *p* < 0.05) and SE (*r* = 0.879, *p* < 0.05) of the participants in this study were highly correlated, only the right eye was included in the analysis. Continuous variables are described using $\bar{x}\pm s$ ,and analysis of variance was used to compare differences between different groups. Categorical data were compared using the $\chi^{2}$ test, and *p* < 0.05 was considered statistically significant.

## Results

A total of 848,697 students from primary and secondary schools in Shenzhen participated in this study. The prevalence rates of myopia in grade 1–9 students in 2019 were 13.6, 19.0, 28.7, 41.0, 52.5, 62.5, 70.9, 77.5, and 81.7%, respectively (Table 2).

**Table 2:** Baseline data of participants in this study in 2019.

Grade at baseline	*N*	Visual acuity	Spherical equivalentrefractions (D)	Prevalence of myopia (%)
Grade 1	155,462	4.93 ± 0.14	−0.12 ± 0.95	13.6
Male	84,119	4.93 ± 0.14	−0.10 ± 0.93	13.1
Female	71,343	4.92 ± 0.14	−0.13 ± 0.96	14.1
Grade 2	150,846	4.92 ± 0.16	−0.32 ± 1.02	19.0
Male	81,357	4.92 ± 0.16	−0.29 ± 1.01	18.2
Female	69,489	4.92 ± 0.16	−0.36 ± 1.02	19.8
Grade 3	129,019	4.89 ± 0.21	−0.58 ± 1.14	28.7
Male	70,040	4.89 ± 0.21	−0.54 ± 1.14	27.0
Female	58,979	4.88 ± 0.22	−0.64 ± 1.13	30.8
Grade 4	107,871	4.83 ± 0.27	−0.90 ± 1.29	41.0
Male	59,055	4.84 ± 0.26	−0.83 ± 1.30	37.8
Female	48,816	4.81 ± 0.28	−0.99 ± 1.28	44.9
Grade 5	94,704	4.76 ± 0.31	−1.26 ± 1.47	52.5
Male	51,898	4.78 ± 0.30	−1.15 ± 1.47	47.8
Female	42,806	4.72 ± 0.32	−1.39 ± 1.45	58.2
Grade 6	83,642	4.68 ± 0.35	−1.65 ± 1.64	62.5
Male	45,958	4.72 ± 0.33	−1.52 ± 1.64	57.2
Female	37,684	4.64 ± 0.35	−1.80 ± 1.62	69.0
Grade 7	62,713	4.60 ± 0.38	−2.05 ± 1.80	70.9
Male	33,821	4.64 ± 0.37	−1.92 ± 1.81	65.9
Female	28,892	4.54 ± 0.38	−2.19 ± 1.77	76.7
Grade 8	41,236	4.52 ± 0.39	−2.47 ± 1.91	77.5
Male	22,254	4.57 ± 0.38	−2.35 ± 1.93	73.1
Female	18,982	4.47 ± 0.39	−2.62 ± 1.88	82.6
Grade 9	23,204	4.47 ± 0.38	−2.80 ± 2.01	81.7
Male	12,481	4.52 ± 0.37	−2.70 ± 2.03	78.3
Female	10,723	4.42 ± 0.38	−2.93 ± 1.98	85.6

**Figure 1:**
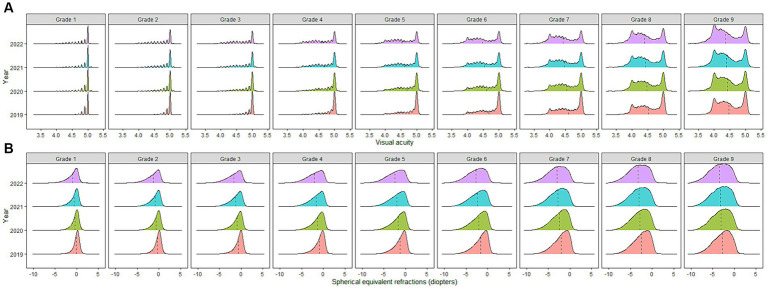
**(A)** Distribution of visual acuity among school-aged children in China from 2019–2022. **(B)** Distribution of spherical equivalent refractions among school-aged children in China from 2019–2022. *The dashed line represents the mean value.

**Figure 2:**
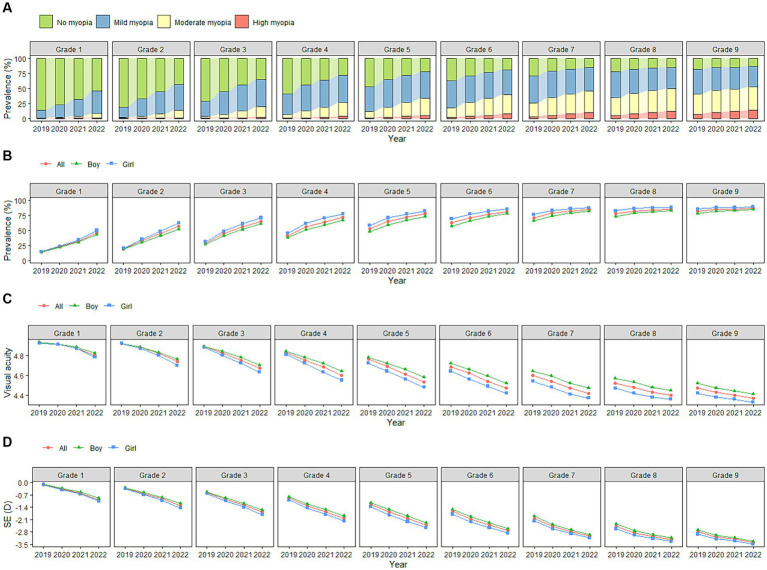
**(A)** Changes in constituent ratio of myopia among school-aged children in China from 2019–2022. **(B)** Changes in myopia prevalence among school-aged children in China from 2019–2022. **(C)** Changes in visual acuity among school-aged children in China from 2019–2022. **(D)** Changes in spherical equivalent refractions among school-aged children in China from 2019–2022.

From 2019 to 2022, visual acuity measurements across all grades followed a downward trend. Over the observation period, the participants showed a decrease of 0.18 ± 0.30 (*p* < 0.001) in uncorrected visual acuity, with boys and girls experiencing a decrease of 0.17 ± 0.29 (*p* < 0.001) and 0.21 ± 0.32 (*p* < 0.001), respectively. Students who were in grade 4 at baseline showed the greatest decline in visual acuity (0.23 ± 0.31, *p* < 0.001), followed by those who were in grades 3 (0.22 ± 0.31, *p* < 0.001) and 5 (0.22 ± 0.31, *p* < 0.001) at baseline. Except for those students who were in grades 7–9 at baseline, boys in the other grades showed lower uncorrected visual acuity decreases than girls. Among the girls, those who were in grade 4 at baseline showed the greatest decrease in visual acuity (0.26 ± 0.33, *p* < 0.001), while among the boys, those who were in grades 4–6 at baseline showed the greatest decrease in visual acuity (0.20 ± 0.29, *p* < 0.001). During the COVID-19 pandemic, the participants’ visual acuity levels decreased by 0.05 ± 0.19 (*p* < 0.001), of which those who were in grades 4–6 at baseline experienced the greatest decline (0.07, *p* < 0.001). A more detailed overview of the results is provided in Table 3 and Figures 1A, 2C.

**Table 3:** Visual acuity progression among school-aged children from 2019–2022.

Grade at baseline	Change from 2019–2022	Annual change			*F*	P*
		2019–2020	2020–2021	2021–2022		
Grade 1	−0.12 ± 0.28	−0.01 ± 0.16	−0.04 ± 0.17	−0.07 ± 0.19	4,632.97	< 0.001
Male	−0.11 ± 0.27	−0.01 ± 0.16	−0.03 ± 0.16	−0.06 ± 0.18	1,911.80	< 0.001
Female	−0.14 ± 0.29	−0.01 ± 0.16	−0.04 ± 0.17	−0.08 ± 0.20	2,793.32	< 0.001
Grade 2	−0.19 ± 0.29	−0.05 ± 0.17	−0.06 ± 0.19	−0.08 ± 0.20	1,036.41	< 0.001
Male	−0.16 ± 0.28	−0.04 ± 0.16	−0.05 ± 0.18	−0.07 ± 0.19	259.37	< 0.001
Female	−0.22 ± 0.31	−0.05 ± 0.17	−0.07 ± 0.20	−0.09 ± 0.22	729.85	< 0.001
Grade 3	−0.22 ± 0.31	−0.06 ± 0.19	−0.07 ± 0.20	−0.08 ± 0.21	322.01	< 0.001
Male	−0.19 ± 0.29	−0.05 ± 0.18	−0.06 ± 0.19	−0.07 ± 0.20	193.66	< 0.001
Female	−0.25 ± 0.32	−0.08 ± 0.20	−0.09 ± 0.21	−0.09 ± 0.23	46.01	< 0.001
Grade 4	−0.23 ± 0.31	−0.07 ± 0.20	−0.07 ± 0.21	−0.08 ± 0.22	84.01	< 0.001
Male	−0.20 ± 0.29	−0.06 ± 0.19	−0.06 ± 0.19	−0.08 ± 0.21	203.11	< 0.001
Female	−0.26 ± 0.33	−0.09 ± 0.22	−0.09 ± 0.22	−0.08 ± 0.23	32.61	< 0.001
Grade 5	−0.22 ± 0.31	−0.07 ± 0.21	−0.08 ± 0.22	−0.08 ± 0.22	67.21	< 0.001
Male	−0.20 ± 0.29	−0.06 ± 0.20	−0.07 ± 0.20	−0.08 ± 0.21	125.46	< 0.001
Female	−0.25 ± 0.33	−0.09 ± 0.22	−0.08 ± 0.23	−0.08 ± 0.23	27.76	< 0.001
Grade 6	−0.21 ± 0.30	−0.07 ± 0.22	−0.07 ± 0.22	−0.07 ± 0.22	0.00	> 0.05
Male	−0.20 ± 0.29	−0.06 ± 0.21	−0.07 ± 0.21	−0.07 ± 0.21	28.96	< 0.001
Female	−0.22 ± 0.32	−0.08 ± 0.23	−0.07 ± 0.23	−0.07 ± 0.23	23.75	< 0.001
Grade 7	−0.17 ± 0.31	−0.06 ± 0.23	−0.07 ± 0.22	−0.05 ± 0.23	122.01	< 0.001
Male	−0.17 ± 0.29	−0.05 ± 0.22	−0.07 ± 0.21	−0.05 ± 0.22	96.01	< 0.001
Female	−0.17 ± 0.32	−0.06 ± 0.24	−0.07 ± 0.23	−0.04 ± 0.25	116.90	< 0.001
Grade 8	−0.12 ± 0.29	−0.05 ± 0.23	−0.05 ± 0.23	−0.02 ± 0.23	233.85	< 0.001
Male	−0.13 ± 0.28	−0.05 ± 0.22	−0.05 ± 0.21	−0.03 ± 0.22	61.31	< 0.001
Female	−0.11 ± 0.30	−0.05 ± 0.24	−0.05 ± 0.24	−0.02 ± 0.24	98.86	< 0.001
Grade 9	−0.10 ± 0.27	−0.05 ± 0.23	−0.03 ± 0.22	−0.03 ± 0.23	62.00	< 0.001
Male	−0.11 ± 0.26	−0.05 ± 0.21	−0.03 ± 0.21	−0.03 ± 0.22	37.74	< 0.001
Female	−0.09 ± 0.28	−0.04 ± 0.24	−0.02 ± 0.23	−0.03 ± 0.24	19.14	< 0.001
Total	−0.18 ± 0.30	−0.05 ± 0.19	−0.06 ± 0.20	−0.07 ± 0.21	2,118.21	< 0.001
Male	−0.17 ± 0.29	−0.05 ± 0.18	−0.06 ± 0.19	−0.07 ± 0.20	1,274.61	< 0.001
Female	−0.21 ± 0.32	−0.06 ± 0.21	−0.07 ± 0.21	−0.08 ± 0.23	824.34	< 0.001

The *p*-values refer to overall comparisons.

From 2019 to 2022, the SE of the participants showed a myopic drift, decreasing by 1.00 ± 1.27 D (*p* < 0.001). Boys and girls dropped by 0.96 ± 1.25 D (*p* < 0.001) and 1.05 ± 1.31 D (*p* < 0.001), respectively. The students who were in grade 5 at baseline showed the highest decrease in SE (1.15 D ± 1.22, *p* < 0.001), followed by those who were in grades 4 (1.13 D ± 1.30, *p* < 0.001) and 3 (1.09 D ± 1.35, *p* < 0.001) at baseline. Except for those who were in grades 6–9 at baseline, the SE decreases of the boys in the other grades were lower than those of the girls. Among the girls, those who were in grade 4 at baseline showed the greatest decrease in SE (1.20 D ± 1.33, *p* < 0.001). Among the boys, those who were in grade 5 at baseline showed the greatest decrease in SE (1.12 D ± 1.22, *p* < 0.001). During the COVID-19 pandemic, the participants’ SE decreased by 0.36 D ± 0.89 (*p* < 0.001), among which the students who were in grade 7 at baseline had the highest decrease (0.45 D ± 0.78, *p* < 0.001). A more detailed overview of the results is provided in Table 4 and Figures 1B, 2D.

**Table 4:** Spherical equivalent refraction progression among school-aged children from 2019–2022.

Grade at baseline	Change from 2019–2022 (D)	Annual change (D)			*F*	P*
		2019–2020	2020 2021	2021–2022		
Grade 1	−0.86 ± 1.33	−0.26 ± 0.96	−0.23 ± 0.88	−0.36 ± 0.85	893.50	< 0.001
Male	−0.80 ± 1.30	−0.24 ± 0.94	−0.22 ± 0.85	−0.34 ± 0.81	461.09	< 0.001
Female	−0.93 ± 1.36	−0.28 ± 0.98	−0.25 ± 0.91	−0.40 ± 0.88	526.12	< 0.001
Grade 2	−0.99 ± 1.35	−0.31 ± 0.94	−0.30 ± 0.87	−0.38 ± 0.81	374.39	< 0.001
Male	−0.91 ± 1.31	−0.29 ± 0.92	−0.27 ± 0.84	−0.35 ± 0.77	197.24	< 0.001
Female	−1.09 ± 1.40	−0.33 ± 0.97	−0.33 ± 0.90	−0.43 ± 0.84	268.57	< 0.001
Grade 3	−1.09 ± 1.35	−0.37 ± 0.91	−0.33 ± 0.83	−0.39 ± 0.78	169.97	< 0.001
Male	−1.01 ± 1.30	−0.35 ± 0.88	−0.30 ± 0.80	−0.37 ± 0.75	138.17	< 0.001
Female	−1.19 ± 1.39	−0.40 ± 0.94	−0.37 ± 0.86	−0.42 ± 0.81	49.16	< 0.001
Grade 4	−1.13 ± 1.30	−0.41 ± 0.88	−0.34 ± 0.79	−0.37 ± 0.75	203.53	< 0.001
Male	−1.06 ± 1.27	−0.38 ± 0.84	−0.31 ± 0.77	−0.37 ± 0.74	137.55	< 0.001
Female	−1.20 ± 1.33	−0.46 ± 0.92	−0.37 ± 0.82	−0.37 ± 0.77	180.45	< 0.001
Grade 5	−1.15 ± 1.22	−0.42 ± 0.83	−0.36 ± 0.76	−0.37 ± 0.72	164.48	< 0.001
Male	−1.12 ± 1.22	−0.39 ± 0.82	−0.35 ± 0.76	−0.38 ± 0.71	38.46	< 0.001
Female	−1.18 ± 1.22	−0.46 ± 0.83	−0.36 ± 0.77	−0.36 ± 0.72	228.34	< 0.001
Grade 6	−1.08 ± 1.14	−0.42 ± 0.81	−0.35 ± 0.76	−0.31 ± 0.69	458.62	< 0.001
Male	−1.08 ± 1.14	−0.40 ± 0.81	−0.36 ± 0.76	−0.32 ± 0.68	130.06	< 0.001
Female	−1.07 ± 1.13	−0.43 ± 0.81	−0.33 ± 0.76	−0.31 ± 0.69	273.30	< 0.001
Grade 7	−0.99 ± 1.11	−0.45 ± 0.78	−0.30 ± 0.71	−0.25 ± 0.72	1249.72	< 0.001
Male	−1.03 ± 1.09	−0.45 ± 0.78	−0.31 ± 0.69	−0.26 ± 0.67	641.84	< 0.001
Female	−0.95 ± 1.13	−0.44 ± 0.77	−0.28 ± 0.73	−0.23 ± 0.77	606.85	< 0.001
Grade 8	−0.76 ± 1.05	−0.38 ± 0.76	−0.20 ± 0.72	−0.18 ± 0.71	938.06	< 0.001
Male	−0.80 ± 1.02	−0.39 ± 0.75	−0.21 ± 0.68	−0.19 ± 0.66	554.64	< 0.001
Female	−0.72 ± 1.09	−0.36 ± 0.76	−0.19 ± 0.76	−0.17 ± 0.75	361.36	< 0.001
Grade 9	−0.60 ± 1.05	−0.30 ± 0.81	−0.10 ± 0.70	−0.21 ± 0.71	423.25	< 0.001
Male	−0.64 ± 1.01	−0.32 ± 0.81	−0.11 ± 0.65	−0.22 ± 0.64	277.60	< 0.001
Female	−0.56 ± 1.09	−0.28 ± 0.81	−0.08 ± 0.76	−0.20 ± 0.79	175.47	< 0.001
Total	−1.00 ± 1.27	−0.36 ± 0.89	−0.30 ± 0.81	−0.35 ± 0.77	1288.99	< 0.001
Male	−0.96 ± 1.25	−0.34 ± 0.87	−0.29 ± 0.79	−0.34 ± 0.74	539.06	< 0.001
Female	−1.05 ± 1.31	−0.38 ± 0.91	−0.31 ± 0.84	−0.36 ± 0.80	695.63	< 0.001

The *p*-values refer to overall comparisons.

During the observation period, the prevalence of myopia among the participants of all grades showed an increasing trend. It increased by 28.2% overall, by 27.0 and 29.8% in boys and girls (*p* < 0.001), respectively, showing a statistically significant difference. The students who were in grade 2 at baseline had the highest increase (37.6%, *p* < 0.001), followed by those who were in grades 3 (36.6%, *p* < 0.001) and 1 (32.4%, *p* < 0.001) at baseline. During the COVID-19 pandemic, the prevalence of myopia among the participants increased by 11.3% (10.6% for boys and 12.2% for girls), with the highest increase (15.7%) seen in those who were in grade 3 at baseline. Detailed results are shown in Table 5 and Figure 2B. Further analysis showed that as the grade increased the proportion of mild myopia increased first, followed by a decrease—both of which were statistically significant (*p < 0.05*). With increasing grades, the proportion of severe and moderate myopia also increased, showing a statistically significant difference (*p* < 0.05) (Table 6 and Figure 2A).

**Table 5:** Prevalence of myopia among school-aged children from 2019–2022.

Grade at baseline	Change of prevalence from 2019–2022 (%)	Prevalence (%)				$\chi^{2}$	P*
		2019	2020	2021	2022		
Grade 1	32.4	13.6	22.5	31.8	46.0	43,901.76	< 0.001
Male	29.8	13.1	21.8	30.2	42.9	20,626.44	< 0.001
Female	35.6	14.1	23.4	33.8	49.7	23,623.25	< 0.001
Grade 2	37.6	19.0	32.2	44.6	56.6	50,153.44	< 0.001
Male	33.9	18.2	30.1	41.2	52.1	22,642.81	< 0.001
Female	42.2	19.8	34.7	48.5	62.0	28,276.03	< 0.001
Grade 3	36.6	28.7	44.4	55.6	65.3	38,301.51	< 0.001
Male	33.6	27.0	40.9	51.3	60.6	17,657.91	< 0.001
Female	40.0	30.8	48.5	60.8	70.8	21,070.14	< 0.001
Grade 4	30.7	41.0	55.7	64.2	71.7	23,064.20	< 0.001
Male	29.4	37.8	51.0	59.1	67.2	11,201.33	< 0.001
Female	32.3	44.9	61.4	70.4	77.2	12,331.63	< 0.001
Grade 5	24.9	52.5	64.3	71.4	77.4	14,586.11	< 0.001
Male	25.5	47.8	58.9	66.5	73.3	7,870.26	< 0.001
Female	24.1	58.2	70.9	77.2	82.3	6,916.91	< 0.001
Grade 6	18.8	62.5	71.2	76.8	81.3	8,342.17	< 0.001
Male	20.4	57.2	66.1	72.7	77.6	4,972.49	< 0.001
Female	16.7	69.0	77.4	81.9	85.7	3,457.71	< 0.001
Grade 7	13.6	70.9	78.5	82.3	84.5	4,051.49	< 0.001
Male	16.0	65.9	74.4	78.8	81.9	2,632.59	< 0.001
Female	11.0	76.7	83.3	86.3	87.7	1,504.84	< 0.001
Grade 8	7.9	77.5	82.3	84.2	85.4	1,028.43	< 0.001
Male	9.8	73.1	78.6	81.2	82.9	736.62	< 0.001
Female	5.8	82.6	86.7	87.7	88.4	325.47	< 0.001
Grade 9	5.1	81.7	85.1	85.3	86.8	249.71	< 0.001
Male	6.6	78.3	82.3	83.1	84.9	198.40	< 0.001
Female	3.5	85.6	88.3	88.0	89.1	67.93	< 0.001
Total	28.2	38.7	50.0	58.4	66.9	148352.68	< 0.001
Male	27.0	36.1	46.7	54.8	63.1	73505.10	< 0.001
Female	29.8	41.7	53.9	62.7	71.5	77024.24	< 0.001

The *p*-values refer to overall comparisons.

**Table 6:** Proportion of varying degrees of myopia among school-aged children from 2019–2022.

Grade at baseline	2019 (%)				2020 (%)				2021 (%)				2022 (%)			
	No myopia	Mild myopia	Moderate myopia	Severe myopia	No myopia	Mild myopia	Moderate myopia	Severe myopia	No myopia	Mild myopia	Moderate myopia	Severe myopia	No myopia	Mild myopia	Moderate myopia	Severe myopia
Grade 1	86.4	12.7	0.7	0.1	77.5	20.9	1.5	0.1	68.2	28.0	3.6	0.2	54.0	37.9	7.6	0.5
Grade 2	81.0	17.6	1.2	0.1	67.8	28.6	3.4	0.2	55.4	37.4	6.7	0.4	43.4	43.6	12.1	1.0
Grade 3	71.3	25.7	2.9	0.1	55.6	37.4	6.6	0.4	44.4	43.2	11.6	0.9	34.7	45.6	17.8	1.8
Grade 4	59.0	34.8	6.0	0.3	44.3	43.5	11.3	0.9	35.8	45.4	17.1	1.7	28.3	45.3	23.3	3.1
Grade 5	47.5	41.2	10.7	0.6	35.7	45.6	17.1	1.6	28.6	45.1	23.2	3.0	22.6	43.6	28.8	4.9
Grade 6	37.5	44.5	16.6	1.4	28.8	44.8	23.3	3.1	23.2	43.3	28.6	4.9	18.7	41.4	32.8	7.1
Grade 7	29.1	45.0	23.1	2.9	21.5	43.9	29.5	5.0	17.7	41.5	33.7	7.1	15.5	38.4	36.8	9.3
Grade 8	22.5	43.2	29.6	4.7	17.7	40.5	34.4	7.4	15.8	37.9	36.9	9.4	14.6	35.8	38.1	11.6
Grade 9	18.3	40.7	34.0	7.0	14.9	38.1	37.4	9.7	14.7	36.4	37.8	11.2	13.2	34.4	39.0	13.5
$x^{2}$	220,546.22				198,161.70				165,680.18				123,252.56			
P*	≤0.001				≤0.001				≤0.001				≤0.001			

The *p*-values refer to overall comparisons.

We selected students in grade 1 who were not myopic at baseline for follow-up and found that the 3-year cumulative incidence of myopia of students with baseline SE of ≥1.00 D, ≥ 0.50 D and < 1.00 D, ≥ 0 D and < 0.50 D, and ≥ −0.50 D and < 0 D were 6.8, 24.8, 39.0, and 48.1%, respectively. Detailed results are presented in Table 7.

**Table 7:** Cumulative incidence of myopia among students in Grade 1 with different baseline spherical equivalent refraction.

Baseline SE (D)	1-year follow-up (%)	2-year follow-up (%)	3-year follow-up (%)
−0.5 ≤ Baseline SE < 0 (*n* = 34,327)	24.6	34.3	48.1
0 ≤ Baseline SE < 0.5 (*n* = 51,926)	12.9	23.9	39.0
0.5 ≤ Baseline SE < 1 (*n* = 28,391)	4.5	11.6	24.8
Baseline SE ≥ 1 (*n* = 7,901)	1.6	2.4	6.8
χ^2^	6,565.52	6,586.15	6,836.07
*P*	< 0.001	< 0.001	< 0.001

## Discussion

This four-year cohort study focused on uncorrected visual acuity, SE, and the incidence and prevalence of myopia among school-aged children in Shenzhen, China. During the observation period, it was found that the uncorrected visual acuity and SE measurements of the study participants decreased by 0.18 and 1.00 D, respectively, and that the prevalence of myopia increased by 28.2%. The students who were in grade 4 at baseline showed the greatest decrease in visual acuity (0.23), those who were in grade 5 at baseline showed the greatest decrease in SE (1.15 D), and the prevalence of myopia increased the most among the students who were in grade 2 at baseline (37.6%). Girls showed a greater decrease in visual acuity and SE, and a greater increase in the prevalence of myopia compared to boys of the same grades. For non-myopic students in grade 1 at baseline, the cumulative incidences of myopia in the first, second, and third years of follow-up were 13.5, 22.6, and 36.2%, respectively.

In 2020, many countries around the world—including China—adopted home quarantine policies to prevent and control the COVID-19 pandemic (9). At-home quarantining proved to be an effective way to control the COVID-19 pandemic (19). China suspended schools nationwide from February to May of 2020, with students studying at home or undergoing online learning (10). Specifically, schools were suspended between February and May 2020 as part of the initial response to the COVID-19 outbreak. In addition to the school closures, Shenzhen experienced several other public health measures aimed at controlling the spread of the virus. These included: Before COVID-19 (2019): Regular schooling and activities were uninterrupted, with no special public health measures related to COVID-19. During COVID-19 (2020): Significant disruptions occurred due to the strict lockdown and school closures, with a shift to remote learning and limited physical interactions. After COVID-19 (2021–2022): A gradual return to normalcy was observed, but some public health measures persisted to prevent new outbreaks.

COVID-19 has had a negative impact on health-related behaviors in children (20–22). The time children spent watching electronic screens reached a peak, while the time spent outdoors declined significantly. Screen time in school-aged children increased by approximately 30 h/week during the COVID-19 pandemic (23). Chinese children used smartphones for long periods to play video games and browse the internet during the COVID-19 pandemic (24). Students in grades 4–6 often use smartphones for online learning and social media (24). After the COVID-19 outbreak, it became particularly critical to monitor myopia in a timely manner and explore the characteristics of myopia onset, which is helpful for formulating targeted myopia prevention and control strategies in school-aged children. This study spanned the pre-, mid-, and post-pandemic periods, and analyzed the epidemiological characteristics of myopia among school-aged children in China, with a goal of providing a scientific basis for formulating targeted myopia prevention and control strategies in this vulnerable demographic.

We found that the visual acuity measurements of our study participants decreased by 0.18 over four years, and by 0.05, 0.06, and 0.07 over 2019–2020, 2020–2021, and 2021–2022, respectively. The SE decreased by 1.00 D over four years, and by 0.36 D, 0.30 D, and 0.35 D over 2019–2020, 2020–2021, and 2021–2022, respectively. Consistent with the results of other studies (17, 25), the risk of myopia among school-aged children remained high in 2021 and 2022, after the home-quarantine order was lifted. This may be related to failures to change unhealthy lifestyles and habits of eye use that many children developed during the pandemic. Study found that, although children were more involved in health-related behaviors after the pandemic subsided, their participation levels were lower than they were in the pre-COVID-19 period (13). In this study, the SE of the participants during the COVID-19 pandemic decreased by 0.36 D, which was similar to that in Shantou and Guangdong (−0.35 D) (26), and lower than that in Chongqing (−0.43 D) (27), Anyang (−0.46 D) (28), and Wenzhou (−0.44 D) (29).

One survey found that the prevalence of myopia among children and adolescents in nine Chinese provinces increased by 11.7% during the COVID-19 pandemic (30). Similarly, the prevalence of myopia among the students in this study increased by 11.3% during the COVID-19 pandemic. With increasing age, the prevalence of severe myopia gradually increased, and there was no severe myopia observed among preschool children. The prevalence of severe myopia has been reported to increase slowly during primary school, then rapidly during secondary school and high school (31). The prevalence of severe myopia among the participants in this study gradually increased in 2019, 2020, 2021, and 2022, to 0.9, 1.7, 2.6, and 3.8%, respectively. The prevalence of severe myopia is predicted to increase significantly globally, with 9.8% of the global population predicted to suffer from severe myopia by 2050 (1). In this study, the prevalence of severe myopia among the study participants in 2022 was 3.8%, which was higher than that in Shandong (2.0%) (32) and Anyang (1.2%) (33), but lower than that in Beijing (8.6%) (31) and Zhejiang (9.4%) (34). These differences may be caused by multiple factors, including lifestyle, environment, and genetics.

When humans are born, the eyeball is in a hyperopic state, with its SE ranging from +2.50 ~ +3.00 D. Over time, this SE gradually tends toward mild hyperopia or emmetropia. This process is often called “emmetropization” (35), and the degree of hyperopia present in the process is called “hyperopia reserve” (28). Hyperopia reserves in children and adolescents gradually decrease over time. With a decrease in the hyperopia reserve, the cumulative incidence of myopia in primary school students gradually increases over 1–3 years. If students have not yet developed myopia in the first grade of primary school, but are already in the emmetropic state (−0.50 to 0 D), their 3-year cumulative incidence of myopia can be as high as >48%. When the hyperopia reserve reaches above +1.00 D, the 3-year cumulative incidence of myopia is likely to be <7%. Pre-myopia is a key intermediate stage in refractive development, and the pre-myopia stage is a high-risk stage of myopia (36). Therefore, early screening myopia is an effective means to prevent myopia. Children and adolescents use their eyes at close ranges too early and too often, which leads to excessive consumption of hyperopia reserves and increases the likelihood of developing myopia. Therefore, scientific and timely monitoring of hyperopia reserves in children and early detection of children at high risk of developing myopia are important for its prevention and control.

There are some significant advantages to this study. First, its sample size was large, with over 840,000 children and adolescents participating. Second, participant compliance was good, with a loss-to-follow-up rate of only 0.37%. Third, the study population included students of all grades in primary and secondary schools, and the results comprehensively reflected the epidemiological characteristics of myopia in students of all ages. Fourth, this study relied on the Shenzhen Children and Adolescents Myopia Monitoring Big Data Platform, and all data were uploaded to the platform in real time at the screening site, which saved time and effort and provided data-based support for the study. Fifth, based on the baseline, the study participants were followed up continuously for 3 years, which was a long enough period to reveal changes in myopia over time. However, there are some key shortcomings to this study that are worth noting as well. First, although non-cycloplegic refractive examination has been proven to have high sensitivity and specificity for large-scale myopia screening (37), it may overestimate the prevalence of myopia to some extent. Second, some biological parameters of the eyeball that are closely related to refraction, such as the corneal radius (CR) and axial length (AL), were not included as screening indicators. One study found that AL and the AL/CR ratio are closely related to refractive status (38). Third, the age of the subjects in this study was not recorded during follow-up, so age-specific SE changes of the subjects could not be analyzed.

This study revealed changing trends in visual acuity and refractive status during and after the COVID-19 pandemic among school-aged children in China. Over a follow-up period of 4 years, the participants’ visual acuity measurements progressed to −0.18 ± 0.30 D (−0.17 ± 0.29 D for boys, −0.21 ± 0.32 D for girls), their SE progressed to −1.00 ± 1.27 D (−0.96 ± 1.25 D for boys, −1.05 ± 1.31 D for girls), and the prevalence of myopia and severe myopia increased by 28.2 and 2.9%, respectively. During the COVID-19 pandemic, the participants’ visual acuity and SE measurements decreased by 0.05 and 0.36 D, respectively, and the prevalence of myopia increased by 11.3%. As the risk of myopia did not decrease after the at-home quarantine order was lifted, we recommend timely monitoring and early intervention for myopia in school-aged children.

## Data Availability

The original contributions presented in the study are included in the article/supplementary materials, further inquiries can be directed to the corresponding author.
